# Composition and Potency Characterization of *Mycobacterium avium* subsp. *paratuberculosis* Purified Protein Derivatives

**DOI:** 10.1371/journal.pone.0154685

**Published:** 2016-05-02

**Authors:** Randal T. Capsel, Charles O. Thoen, Timothy A. Reinhardt, John D. Lippolis, Renee Olsen, Judith R. Stabel, John P. Bannantine

**Affiliations:** 1 National Veterinary Services Laboratories, U.S. Department of Agriculture-AHPIS, Ames, Iowa, United States of America; 2 Department of Veterinary Microbiology and Preventive Medicine, Iowa State University, College of Veterinary Medicine, Ames, Iowa, United States of America; 3 National Animal Disease Center, U.S. Department of Agriculture-ARS, Ames, Iowa, United States of America; 4 Center for Veterinary Biologics, U.S. Department of Agriculture-APHIS, Ames, Iowa, United States of America; Pacific Northwest National Laboratory, UNITED STATES

## Abstract

*Mycobacterium avium* subsp. *paratuberculosis* (MAP) purified protein derivatives (PPDs) are immunologic reagents prepared from cultured filtrates of the type strain. Traditional production consists of floating culture incubation at 37°C, organism inactivation by autoclaving, coarse filtration, and protein precipitation. Three traditional production PPDs were used in this study including lot 9801, which served as a reference and has been used in the field for decades. Alternative production PPDs (0902A and 0902B), in which the autoclaving step was removed, were also analyzed in this study. SDS-PAGE analysis revealed protein smearing in traditional PPDs, but distinct bands were observed in the alternative PPD preparations. Antibody bound distinct protein bands in the alternative PPDs by immunoblot analysis, whereas an immunoreactive smear was observed with the traditional PPDs. Mass spectrometry identified 194 proteins among three PPD lots representing the two different production methods, ten of which were present in all PPDs examined. Selected proteins identified by mass spectrometry were recombinantly expressed and purified from *E*. *coli* and evaluated by the guinea pig potency test. Seven recombinant proteins showed greater erythema as compared to the reference PPD lot 9801 in paired guinea pigs and were able to stimulate interferon-gamma production in blood from Johne’s positive animals. These results suggest that autoclaving culture suspensions is not a necessary step in PPD production and specific proteins could supplant the PPD antigen for intradermal skin testing procedures and for use as *in-vitro* assay reagents.

## Introduction

Johne’s disease is a chronic disease of cattle, causing major economic losses to the dairy and beef industry. The economic impact of Johne’s disease is estimated to reach into the millions of dollars annually. A United States Department of Agriculture (USDA) study showed a loss of approximately $200 per cow each year with an annual economic loss of between $200 million to $250 million dollars to the U.S. dairy industry [1]. In addition, Johne’s disease can afflict sheep and various ruminant and non-ruminant wildlife species [2, 3], providing additional reservoirs for the disease causing pathogen, *Mycobacterium avium* subsp. *paratuberculosis* (MAP).

An immunologic reagent was developed in the early 1900s for skin testing of cattle as a simple way to determine exposure to MAP. This reagent, referred to as Johnin, consisted of heat-concentrated culture filtrate proteins obtained after long-term MAP culture in defined conditions. Advances to the production process with the addition of a protein precipitation step led to an improved product, termed purified protein derivative or PPD. When PPD was injected into the skin of a cow, a large erythema due to a delayed-type hypersensitivity (DTH) response measured 72 hours later would indicate MAP exposure. Testing methods that involve the use of PPD include skin testing [4, 5] and more recently it has been routinely incorporated as a stimulating antigen in the gamma interferon test for Johne’s disease [6–11].

The National Veterinary Services Laboratories (NVSL) has been involved in *M*. *bovis* PPD and MAP PPD production since the early 1970’s. All production lots must be tested for potency in guinea pigs prior to distribution and use for skin testing cattle. Historical production methods consisted of obtaining floating MAP cultures in Povitsky bottles, which was how the reference field lot 9801 was prepared, but have more recently transitioned to using Erlenmeyer flasks. Distribution of MAP PPD domestically is minor in comparison to *M*. *bovis* PPD and is used in the field on a limited basis. Contributing to the reduced demand for MAP PPD is the requirement for producers to use either serological assays or fecal culture, or a combination of both for determining a herd’s eligibility in the USDA Voluntary Johne’s Disease Control Program, which is no longer funded. With the emphasis on culture and serology as the approved testing methods, the number of U.S. dairy herds skin tested is projected to decrease. In contrast to lower domestic requests, the NVSL has had increased requests for MAP PPD from India and the United Kingdom. Having a consistent, well-characterized MAP PPD as part of an intradermal skin testing program or as an antigen in the IFN-γ assay is important for more accurate detection of preclinical stages of infection [10, 12], and may increase confidence for use of intradermal skin testing.

Proteomic analysis has been conducted on MAP PPD preparations previously [13, 14]. An analysis of PPD products from various laboratories and facilities revealed that the proteomic composition was highly similar and that variability observed was due to production protocols specifying differing MAP strains as well as the age of the MAP cultures at harvest [14]. The identified proteins in each PPD preparation varied greatly which may be related to its complexity, production processes or the MAP isolate used. Since the 1980s, the NVSL has used a single strain of MAP (ATCC 19698) for its PPD production, and that strain has not changed significantly with time due to defined culture limits that are set at five passages. Only six proteins were identified in the USDA NVSL PPD [14], but it is hypothesized that MAP PPD contains hundreds of proteins. Another study did not examine PPD produced from the ATCC 19698 strain [13], but did identify 156 proteins in the MAP 3+5/C strain.

The objectives of this investigation were to expand on the quantity of proteins identified in the NVSL MAP PPD preparations and determine if removal of the heating step improves the reagent. Furthermore, we examined if recombinant proteins could potentially replace PPD. Importantly, NVSL MAP PPD suspensions that have been used in the field for decades were selected for identification of the specific protein components. In addition, proteins were identified in other MAP PPD preps to determine which proteins were consistently detected by mass spectrometry. Specific proteins identified by mass spectrometry were further evaluated using potency and IFN-γ assays to determine reactivity as possible diagnostic reagents. Information from this study can be used toward improved production methods for a well characterized and consistent PPD as well as reveal specific proteins useful for *in-vitro* testing. Use of an improved reagent could also improve cell mediated immune response tests for earlier detection of MAP infected animals.

## Materials and Methods

### Bacteria, PPD Preparation and Source

MAP strain ATCC 19698 was used in all the MAP PPD production processes. PPD lots produced at the NVSL in 1998 (9801), and ten years later in 2008 (0802, 0803A) were produced by traditional production methods involving autoclaving of production flasks containing floating cultures with subsequent protein precipitation using trichloroacetic acid (TCA) as described previously [15, 16]. Lot 9801 currently serves as a reference lot and has been used in the field for cattle skin testing purposes since its production in 1998. Lot 0902 was produced by an alternative production method involving sterile filtration through a 0.2 μm membrane (Millipore) in place of autoclaving followed by protein precipitation using TCA. Lots 0902A and 0902B, which comprised the larger PPD 0902 bulk preparation, were harvested on different days from the same culture inoculum. In production of large PPD batches (lots), multiple concentrated harvest bulks are commonly combined to reduce the number of potency tests conducted and allow for larger final product release volumes. Due to the alternative production process used for PPD 0902 these two smaller bulks were held as reserve on each harvest date to evaluate possible differences in this revised procedure. Purity testing was conducted in accordance with the Code of Federal Regulations, 9CFR part 113.26.

### Protein concentration assay

Protein measurements of the final PPD preparations were conducted utilizing the Pierce microplate BCA protein assay procedure according to the manufacturer’s directions (Thermo Scientific), with one modification. The compatibility reagent contained within the assay kit was not used because there were no interfering compounds present within the PPD preparations. Assay plates were read on a VersaMax microplate reader at a wavelength of 562 nm. Protein concentrations were calculated using SoftMax Pro software from a standard curve of bovine serum albumin protein standards.

### SDS-PAGE and immunoblot analysis

Pre-cast 4–12% Novex Bis-Tris gels (Invitrogen, 12 well, 1 mm thickness) or precast 10% Novex Bis-Tris gels (Invitrogen, 12 well, 1 mm thickness) were used for SDS-PAGE separation of PPD lots. MAP PPDs, *M*. *bovis* PPD, and *Mycobacterium avium* subsp *avium* (*M*. *avium*) PPD were standardized to approximately 1.0 mg/ml before loading 15 μg/well onto SDS-PAGE gels. MAP PPD’s were prepared by the addition of 175 μl of sample buffer (4x NuPage LDS Sample Buffer, ThermoFisher Scientific) to 325 μl of PPD for each 500 μl of preparation and then heated at 70°C for ten minutes before loading onto gels.

Electrophoresis was conducted in an Invitrogen XCell SureLock Mini-Cell system at a constant current of 125 mA for 35 minutes. Invitrogen SeeBlue Plus2 prestained molecular weight standards served as markers for molecular weight determination. Gels were stained with Invitrogen Novex Colloidal Blue Stain according to the manufacturer’s directions.

Pre-cast 4–12% Novex Bis-Tris gels (Invitrogen, 1 well, 1-mm thickness) were used for immunoblot analysis. A single, long well was loaded with 500 μl of reduced sample to provide an even distribution of antigen across the entire gel. Electrophoresis was conducted in an Invitrogen XCell SureLock Mini-Cell system at a constant current of 125 mA for 35 minutes.

Electrophoretic transfer of proteins onto nitrocellulose was performed using the Invitrogen XCell II Blot Module and Invitrogen NuPAGE transfer buffer at 160 mA for 1 h. After transfer, membranes were blocked with Sigma Western Blocker solution at room temperature for 1–2 hours with rocking. Membranes were washed three times with Trizma-Buffered Saline plus 0.5% Tween 20 (TBS-T). Cattle sera were diluted 1:20 in Phosphate-Buffered Saline plus 0.5% Tween 20 (PBS-T) and incubated with the membranes at room temperature on a rocker platform for approximately 60 min. Membranes were washed three times with TBS-T.

Membranes were incubated for approximately 60 minutes at room temperature on a rocker with peroxidase labeled rabbit anti-bovine IgG (Jackson Immunoresearch Affini-Pure) diluted 1:2000 in PBS-T. Membranes were then washed three times with TBS-T. Membranes were developed in Sigma TMB Substrate according to the manufacturer’s directions.

### Cattle Sera

Whole blood samples were obtained from cattle housed at the NVSL and also from naturally infected animals on dairy farms located around the US. The dairy farm blood samples were collected by herd veterinarians from traditional dairy cow breeds managed within privately owned herds. Herd owner consent was given for participation in sample collection for later use by the NVSL. Blood was collected via jugular puncture using standard animal welfare handling methods into blood collection bags and later processed for serum. Sera from these animals were used in the annual nationwide NVSL Johne’s disease serology proficiency test program, and thus represent a well-characterized and standardized serum set. Naturally infected cattle were confirmed serologically positive on both the Parachek^®^ (ThermoFisher Scientific) and IDEXX MAP ELISA assays (Table 1). Positive cattle were also confirmed positive by fecal culture on Herrold’s Egg Yolk with Mycobactin J (HEY-MJ). Negative sera were from negative control source animals within the NVSL and also from MAP negative confirmed production herds participating in the USDA Voluntary Bovine Johne’s Disease Control Program. Negative animals were confirmed by bacterial culture and serology using the same diagnostic tests as the positive cattle.

**Table 1:** Cattle Sera used in this study.

Lane in Fig 2	Animal ID	Serological Status^a^	IDEXX ELISA Value	Prionics ELISA Value	Fecal Culture Status^b^
1	Lindenhoff 90 (PA)	Pos	0.712	0.465	Moderate
2	Meyer 1688 (PA)	Pos	1.895	1.34	Moderate
3	Calf1 (IA)	Pos	2.233	1.599	Neg
4	$900 (IA)	Pos	2.55	1.073	Moderate
5	1266SS CA (CA)	Pos	1.914	0.749	High
6	2075 (PA)	Pos	1.938	1.681	High
7	874 (MN)	Pos	1.565	0.822	Neg
8	34 (MN)	Pos	2.875	3.443	Low
9	Horst 256 (PA)	Pos	1.046	0.5125	High
10	834 (IA)	Pos	1.049	0.234	Low
11	E22 (IA)	Pos	1.92	1.28	Moderate
12	Charity (WI)	Pos	2.146	2.975	Low
13	Cow 5 (IA)	Pos	1.506	0.979	High
14	Meyer 70 (PA)	Pos	2.57	2.982	Low
15	Lindenhoff 90 (PA)	Pos	0.712	0.465	Moderate
16	Lizzie (IA)	Neg	0.006	0.036	Neg
17	1823 (IA)	Neg	0.158	0.089	Neg
N/A^c^	790	Neg	0.189	N/A	Neg
N/A^c^	2407	Neg	0.260	N/A	Neg
N/A^c^	2222	Pos	2.32	N/A	Moderate
N/A^c^	8339	Pos	1.93	N/A	Low
N/A^c^	1211	Neg	0.036	N/A	Neg
N/A^c^	1044	Neg	0.079	N/A	Neg
N/A^c^	4	Neg^d^	N/A	N/A	Neg
N/A^c^	11	Neg^d^	N/A	N/A	Neg
N/A^c^	1081	Neg	0.036	N/A	Neg
N/A^c^	212	Neg	0.067	N/A	Neg
N/A^c^	61	Neg^d^	N/A	N/A	Neg
N/A^c^	1392	Neg^d^	N/A	N/A	Neg

^a^ Serological status determined by IDEXX and Parachek ELISA test results.

^b^ 1–10 CFU/gr feces = low shedder; 11–50 CFU/gr feces = moderate shedder; >50 CFU/gram of feces = high shedder

^c^ Serum samples used in IFN-γ testing only.

^d^ Based upon previous IFN-γ testing.

### Recombinant Protein Production

The cloning, production, and purification of recombinant MAP proteins was performed as previously described [17]. Briefly, maltose binding protein (MBP) fusions of MAP predicted coding sequences were constructed in *Escherichia coli* DH5α using the pMAL-c2 vector (New England Biolabs, Beverly, MA). Primers designed from the reading frame of each coding sequence contained an *Xba*I site in the 5’ primer and a *Hind*III site in the 3’ primer for cloning. Ligation of the DNA fragments was followed by transformation into ClearColi BL21(DE3) (Lucigen) and selection on Luria-Bertani (LB) agar plates containing 100 μg/ml ampicillin. PCR screening of ampicillin resistant colonies was conducted with primers used to amplify the sequence from MAP DNA.

Each MBP fusion protein was overexpressed in *E*. *coli* by induction of 1-L LB broth cultures with 0.3 mM isopropyl-β-D-thiogalactopyranoside (IPTG, Sigma Chemical Company, St. Louis, MO) for 2.5 hr with shaking at 37°C. Cells were harvested by centrifugation and resuspended in column buffer for sonication. Affinity chromatography purified protein fractions were eluted and analyzed using a NanoDrop spectrophotometer at 280 nm. The most concentrated fractions were pooled and dialyzed at 4°C against 1.5 L phosphate buffered saline. Purified proteins were finally aliquoted and stored at -20°C for future use.

### Mass Spectrometry

Proteomics experiments were accomplished using previously published methods [18]. Total protein concentrations were determined using the Biorad Protein Assay Kit (BioRad, Hercules CA), along with a known BSA standard. Briefly, 100 μg of each sample was resuspended in 250 μl PBS, 3 μl of 10% sodium deoxycholate acid, and 1 μl of 2% SDS. Samples were heated at 90°C for 20 minutes, then cooled on ice for 5 minutes. Next, 2 μl of 50 mM tris-(2-carboxyethyl) phosphine and 1.5 μl fresh 1M iodoacetimide were added and the samples were incubated 1 hour in the dark at room temperature. An equal volume of methanol was added to the protein sample. Proteins were then digested using 10 μg of trypsin gold (Promega) and incubated overnight at 37°C. Formic acid (10 μl) was added, the sample vortexed, and then centrifuged (14,000 x g for 10 minutes at 4°C) to precipitate detergents. Supernatants were transferred to clean tubes and then dried in a vacuum centrifuge. Peptides were dried in one tube, and held at -80°C until use.

Samples were injected onto nano-LC chromatography using a Proxeon Easy-nLC (ThermoFisher Scientific, West Palm Beach, FL) connected to the mass spectrometer. The chromatography used a trapping column (Proxeon Easy-Column, 2 cm, ID 100 μm, 5um, 120A, C18) and an analytical column (Proxeon Easy-Column, 10 cm, ID 75 μm, 3um, 120A, C18). The gradient using a mobile phase A (95% H2O: 5% acetonitrile and 0.1% formic acid) and mobile phase B (5% H_2_O: 95% acetonitrile and 0.1% formic acid). The gradient was: 0% B for 3 minutes, 0%-8% B from 3–5 minutes, 8–18% B from 5–85 minutes, 18–30% B from 85–100 minutes, 30–90% B from 100–105 minutes, and held at 90% B from 105–120 minutes at continuous flow rate throughout the gradient of 300 nl/min.

The analytical column was connected to a PicoTip Emitter (New Objectives, Woburn, MA; FS360-75-15-N-20) cut to size. The column and Emitter were attached to a LTQ OrbiTrap Velos Pro (ThermoFisher Scientific, West Palm Beach, FL) mass spectrometer using the Proxeon Nanospray Flex Ion Source. The capillary temperature was set at 275°C and spray voltage was 2.8 kV. The mass spectrometer used a data dependent method. In MS mode the instrument was set to scan 300–2000 m/z with a resolution of 30,000 FWHM. A minimal signal of 20,000 could trigger MSMS and 10 consecutive MSMS were possible. The activation type used was HCD. The normalized collision energy was set to 35 and repeat mass exclusion was set to 120 seconds.

Tandem mass spectra were extracted and charge state deconvoluted by Proteome Discoverer version 1.4.0.288 (ThermoFisher Scientific, San Jose, CA, USA; version 1.3.0.339). All MS/MS samples were analyzed using Sequest assuming digestion with trypsin. Sequest was set up to search a combined FASTA database of *Mycobacterium avium* complex (taxon #120793) and *Mycobacterium tuberculosis* complex (taxon #77643) databases generated in July of 2013. Sequest was searched with a fragment ion mass tolerance of 0.800 Da and a parent ion tolerance of 10.0 PPM.

### Guinea Pig Potency Testing of PPDs

Conventional guinea pigs weighing between 450 and 600 grams were paired into cages and acclimated for 1 week to the National Centers for Animal Health vivarium housing conditions prior to testing. Guinea pigs were sensitized by injecting a Johne’s bacterin preparation (NVSL lot #0601) consisting of 23 grams, wet weight, of heat-killed MAP cells homogenized in 180 ml of mineral oil.

Prior to inoculation, the Johne’s bacterin was further diluted 1:4.55 in mineral oil and held at 45°C on a hotplate. Each guinea pig was inoculated intramuscularly with 0.1 ml of the warmed suspension. Control guinea pigs were inoculated intramuscularly with 0.1 ml of phosphate chloride buffer. The sensitization period after inoculation was 35 days after which PPD lot numbers 9801, 0802, 0803A, 0902A were prepared for animal inoculation. Each preparation was diluted to 10 ug/ml and 2 ug/ml in phosphate chloride buffer (34 mM Na_2_HPO_4_, 86 mM NaCl).

After the sensitization period and prior to inoculation all guinea pigs had both sides shaved. The shaved area was divided into two equal blocks. Each block contained four randomized injection sites resulting in eight total injections. This resulted in replicate injections of each PPD in each guinea pig. Guinea pigs were injected intradermally with a volume of 0.1 ml of the designated MAP PPDs. No adverse affects resulted from the procedures used and animals were monitored daily by the animal care staff.

A total of 12 guinea pigs (6 guinea pigs for each PPD concentration) were used for PPD inoculation. An additional 4 guinea pigs were used as negative non-sensitized controls. Twenty-four hours post-injection, test responses were measured to the closest square millimeter by measuring the greater and lesser dimensions of the erythema.

All animal work was approved by the NVSL Institutional Animal Care and Use Committee under protocol numbers 2202 and 2738.

### Guinea Pig Potency Testing of Recombinant Proteins

Forty-seven MAP proteins identified by mass spectrometry were recombinantly expressed in *E*. *coli* for further evaluation in the guinea pig potency assay. Eighteen guinea pigs, sensitized as described previously, were used in the screening of selected proteins.

After the 35-day sensitization period and prior to inoculation, all 16 guinea pigs had both sides shaved. There was a single row of four injections sites blocked out on each side of each animal. This allowed for the injection of 6 proteins and two control proteins in each animal. Seven pair of guinea pigs each received six proteins (42 proteins total) and the final pair of animals received five proteins. One guinea pig in each pair also received either PPD 9801 or PPD 0902, whereas the second guinea pig received the MBP/LacZ control peptide diluted to 10 μg/ml in phosphate chloride buffer. This configuration enabled each of the 47 recombinant proteins to be injected twice. Guinea pigs were injected intradermally with a 0.1-ml volume of the designated antigen.

Twelve recombinant proteins were evaluated further in a second guinea pig potency test. Thirty guinea pigs were sensitized as previously described and four guinea pigs served as non-sensitized control animals. Recombinant proteins were randomized and assigned to respective guinea pig groupings. PPD 9801 served as a positive control and phosphate chloride buffer served as a negative control. Suspensions were randomized utilizing a Microsoft Excel randomization program. Sensitized guinea pigs were assigned to four groups of five animals per group.

After the sensitization period and prior to inoculation all guinea pigs had both sides shaved as before. Each side contained four randomized injection sites resulting in eight total injections for each animal. Each sensitized animal received replicate inoculations of two recombinant proteins divided between the left and right sides. Each animal also received one inoculation of lot 9801 and one inoculation of phosphate chloride buffer. Paired non-sensitized control animals received one inoculation of designated recombinant proteins as well as control suspensions. Guinea pigs were injected intradermally with a 0.1-ml volume of the designated MAP recombinant protein or control suspension. Guinea pigs were housed with 5 animals per cage with each of the five animals from different treatment groups within a cage. Test responses were again measured to the closest square millimeter 24-h post injection. Recombinant protein measurements were compared against the positive control within each animal.

### Gamma-interferon Testing Using Recombinant Proteins

Evaluation of recombinant MAP proteins was conducted using blood from 6 Johne’s positive cattle, 2 *M*. *bovis* sensitized cattle, and 4 non-infected control cattle. Approximately 40–50 ml of blood were collected from each animal into vacutainer blood tubes containing sodium heparin. Blood samples were held at room temperature prior to stimulation.

Between 6–7 hours after collection, blood tubes for each individual animal were mixed well and combined into a sterile 50 ml conical tube. For each animal, 1.5 ml aliquots of whole blood were dispensed into each well of a 24-well tissue culture plate. Blood was cultured either alone as non-stimulated controls or with pokeweed at a concentration of 10 μg/ml to serve as a positive control. Whole blood was also cultured with designated mitogens at 10 μg/ml. Mitogens used for evaluation were as follows: MAP PPD 9801 (PPD-J), *M*. *bovis* PPD (PPD-B), *M*. *avium* PPD (PPD-A), MAP_1997, MAP_4143, MAP_1138c, MAP_3567, MAP_3061c, MAP_2121c, and MAP_3651c. For all mitogens, 100 μl was dispensed into each designated well and each mitogen was cultured in duplicate wells.

Plates were covered and incubated at 37°C for 21–22 hours. Plates were centrifuged at 500 x g for 15 minutes and the plasma from each well was harvested and refrigerated. Plasma samples were tested for IFN-γ production over the next 72 hours.

The IFN-γ ELISA was conducted according to the manufacturer’s directions (BOVIGAM^®^, ThermoFisher Scientific). Samples from each well were incubated in duplicate for one hour at room temperature in a 96-well plate that is pre-coated with anti-bovine IFN-γ antibody. Plates were washed six times with wash solution and then incubated with an anti-bovine IFN-γ-horseradish peroxidase conjugate for 30 minutes at room temperature. Plates were again washed six times with wash solution and incubated with peroxidase substrate for 30 minutes at room temperature. Enzyme stopping solution was added to each well and the plates were read at A_450_ and A_650_ nm.

### Statistical Analysis

The PPD mean skin test responses from the guinea pig potency test were compared by ANOVA. Differences were considered statistically significant at a P value of < 0.05.

Results for the recombinant protein guinea pig potency testing were evaluated by calculating the treatment group mean erythema value using the measurements for each individual animal comprising the treatment group. Paired comparisons calculating the difference between the response of the treatment to the response of the positive control for each animal were completed. The five injections (same protein) were then averaged and ranked based on the average differences. A positive difference indicates the average response from the treatment is higher than the average response from the positive control. For this difference data a 95% confidence interval was also calculated for each treatment group.

## Results

### PPD Potency Testing in guinea pigs

Potency testing of the traditional and alternate PPD production lots resulted in significant differences (*P* < 0.05) when compared against the reference standard (lot 9801; Table 2). Traditional lot 0803A and alternate lot 0902 resulted in the greatest skin test responses and all responses showed a larger erythema as PPD concentration increased. The average area of the erythema at the injection sites for lot numbers 0803A and 0902 were much greater than the average area for the reference standard (261% and 177%, respectively,). Traditional PPD lot 0802 resulted in a measurement that was 79% of the average area compared to the reference standard. An acceptable potency test result for new production lots must have a response that is a minimum of 75% compared to the reference response. Using these criteria, all three PPD lots demonstrated acceptable potency, although 0802 is marginal. These data suggest that the potency was not adversely affected by removal of the autoclave step.

**Table 2:** Guinea pig potency skin test responses at 24 hours post-inoculation of MAP PPD.

PPD Concentration	PPD 9801	PPD 0802	PPD 0803A	PPD 0902
10 ug/ml	143.22 ± 48.82	93.63 ± 15.59	264.25 ± 39.72	202.07 ± 39.63
2 ug/ml	45.52 ± 32.13^a^	53.45 ± 15.49	228.41 ± 41.91	132.50 ± 67.79

^a^ PPD 9801, 2 ug/ml sample data, contained one inoculation of one guinea pig which had no measureable skin reaction. This resulted in an elevated standard deviation value. All guinea pigs were sensitized with MAP Strain 19698 killed cells.

Values represent the average response (mm^2^ ± standard deviation) of six guinea pigs used for evaluating each PPD concentration.

### SDS-PAGE analysis of PPD from different production methods

**Fig 1:**
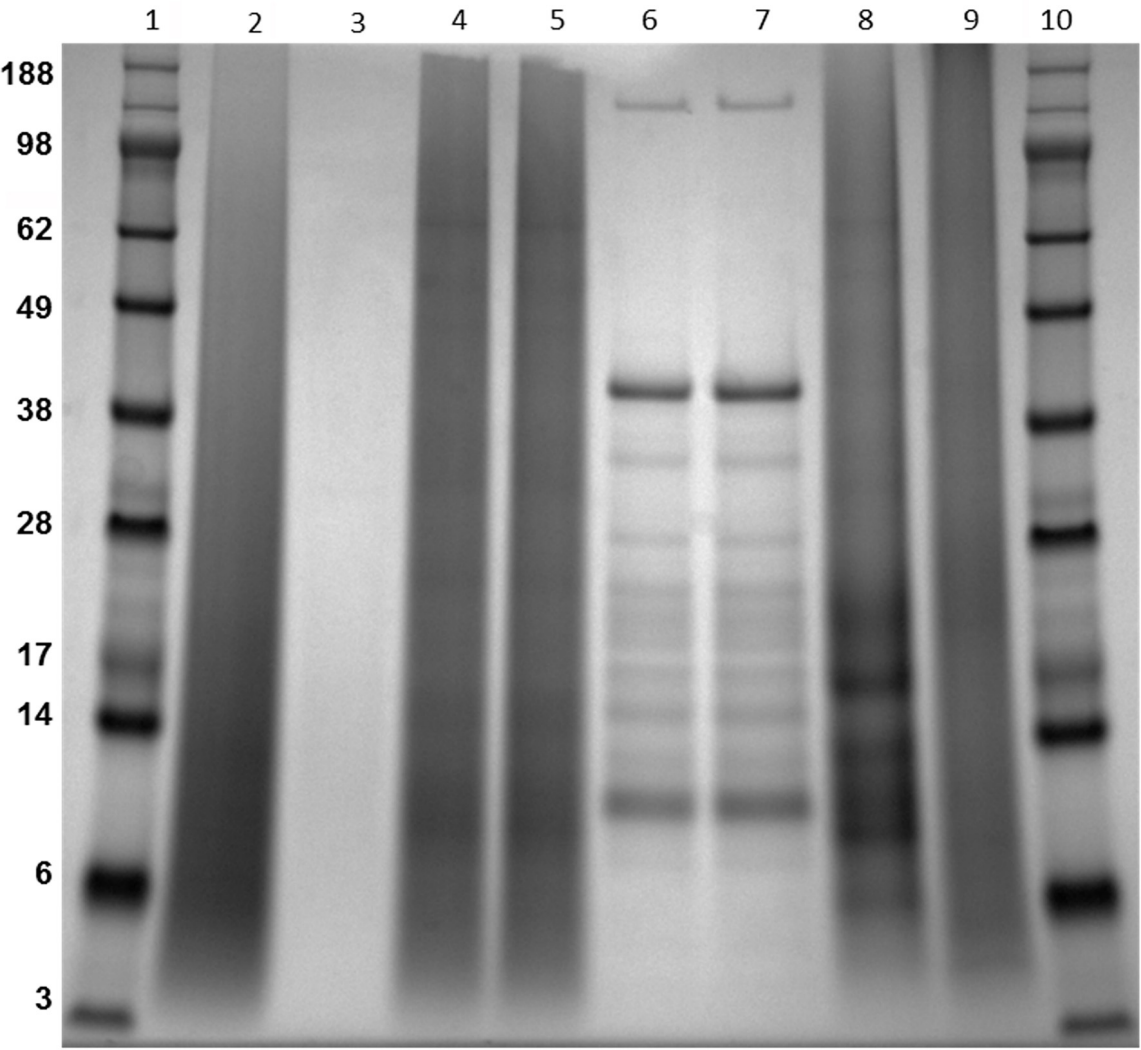
Coomassie blue stained Bis-Tris gel showing 7 PPD preparations. Note the striking differences in traditional production PPDs (lanes 2, 4 and 5 for example) in comparison to the alternative production method (lanes 6 and 7). Lane assignments include: 1 and 10-protein standards; 2-Lot #9801 PPD; 3-Lot 9801 at a 1:10 dilution; 4-Lot 0802; 5-Lot 0803A; 6-Lot 0902A; 7-Lot 0902B; 8-*M*. *bovis* PPD; 9 –*M*. *avium* PPD. Molecular mass standards are indicated in the left margin in kilodaltons.

Aberrant migration or protein smearing was observed by SDS-PAGE analysis of reference PPD Lot 9801; and this resulted in the lack of any discernable protein bands (Fig 1). This result is similar to that observed in other laboratories studying *M*. *bovis* and *M*. *tuberculosis* PPDs [19, 20]. Smearing was also observed on lanes containing lots 0801A, 0801B, 0802, and 0803A from the traditional production method (Fig 1). Similar protein smearing characteristics have also been noted in *M*. *bovis* and *M*. *avium* PPD preparations at the NVSL (unpublished data). The appearance of these “dirty” gel tracks (Fig 1) suggest the autoclaving step may have contributed to this unusual migration pattern, because PPDs produced by the alternate production method each yielded distinct protein bands (Fig 1, lanes 6 and 7). Serial dilution of PPD lot 9801 also failed to display distinct protein bands (Fig 1), suggesting that the smearing was not due to overloading. Protein bands less than 28 kDa were observed in the *M*. *bovis* PPD despite significant smearing, but no protein bands were observed in the *M*. *avium* PPD preparation (Fig 1). Collectively, these data suggest autoclaving modified this reagent by denaturation and/or hydrolysis.

### Humoral immune response against PPD preparations

**Fig 2:**
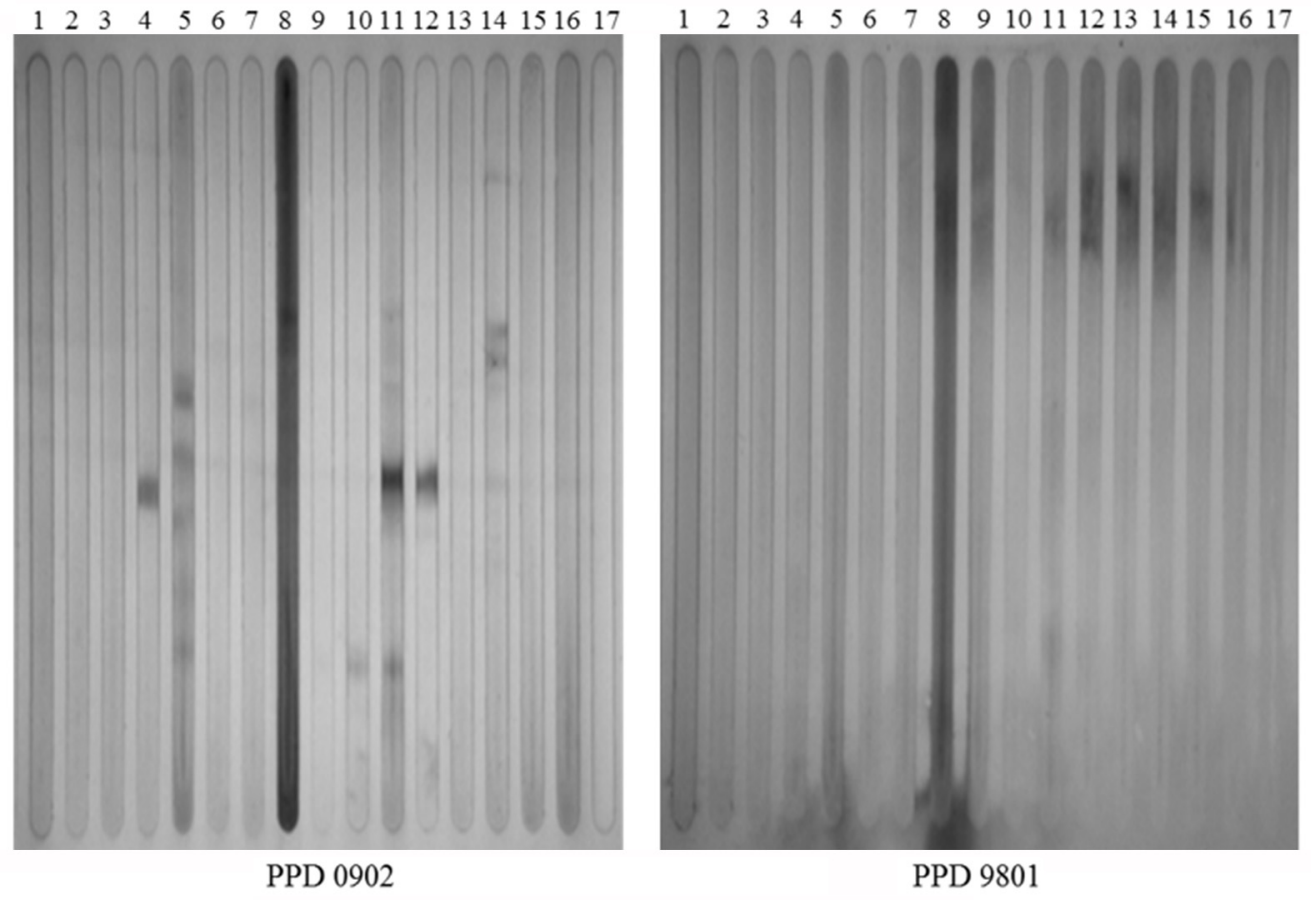
Comparative antibody responses between the PPD production methods. Immunoblots of PPD 0902 produced by an alternative production method on the left and PPD 9801 produced by the traditional method on the right. Both blots were exposed to identical bovine serum samples loaded into independent slots. Note that distinct antigenic bands appear in the alternate PPD preparation. Lanes 1–15 cattle positive for Johne’s disease, Lanes 16 & 17 cattle negative for Johne’s disease. Lane numbers correspond to cattle listed in Table 1.

The affect of autoclaving on the antigenicity of PPDs was next examined. Immunoblots using cattle sera (Table 1) yielded distinct differences between lot 9801 and lot 0902. Immunoreactive protein bands were present in lot 0902, but absent in the reference lot 9801 (Fig 2). Serum from cow 34 in lane 8 had the highest antibody titer to MAP (Table 1) and reactivity was predominant throughout regardless of the PPD production method. However, other sera readily detected protein bands in the alternate PPD but not the traditional PPD. At least one protein band was observed in the alternate PPD from 7 of 15 positive cattle sera. Lanes 4, 11, and 12 containing alternate PPDs all appear to react with a protein of similar size (Fig 2). These results suggest that removing the autoclaving step in the production process may preserve antigenic properties.

### Analysis of PPD preparations by Mass Spectrometry

**Fig 3:**
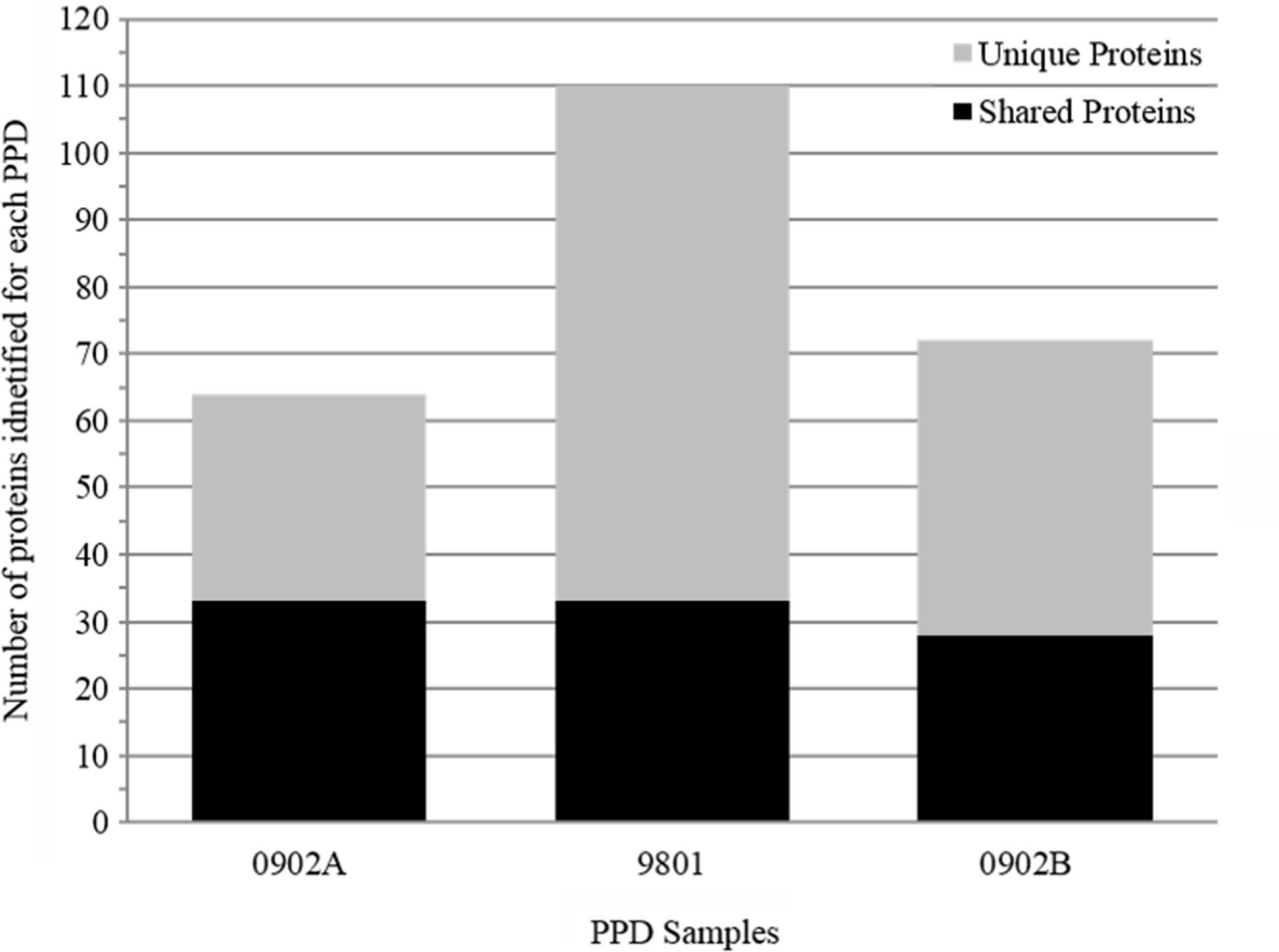
Number of proteins identified by LC-MS/MS for each PPD preparation analyzed. Proteins found in two or three PPD preparations are indicated by the black bars and proteins unique to each individual PPD preparation are indicated by the grey bars.

**Fig 4:**
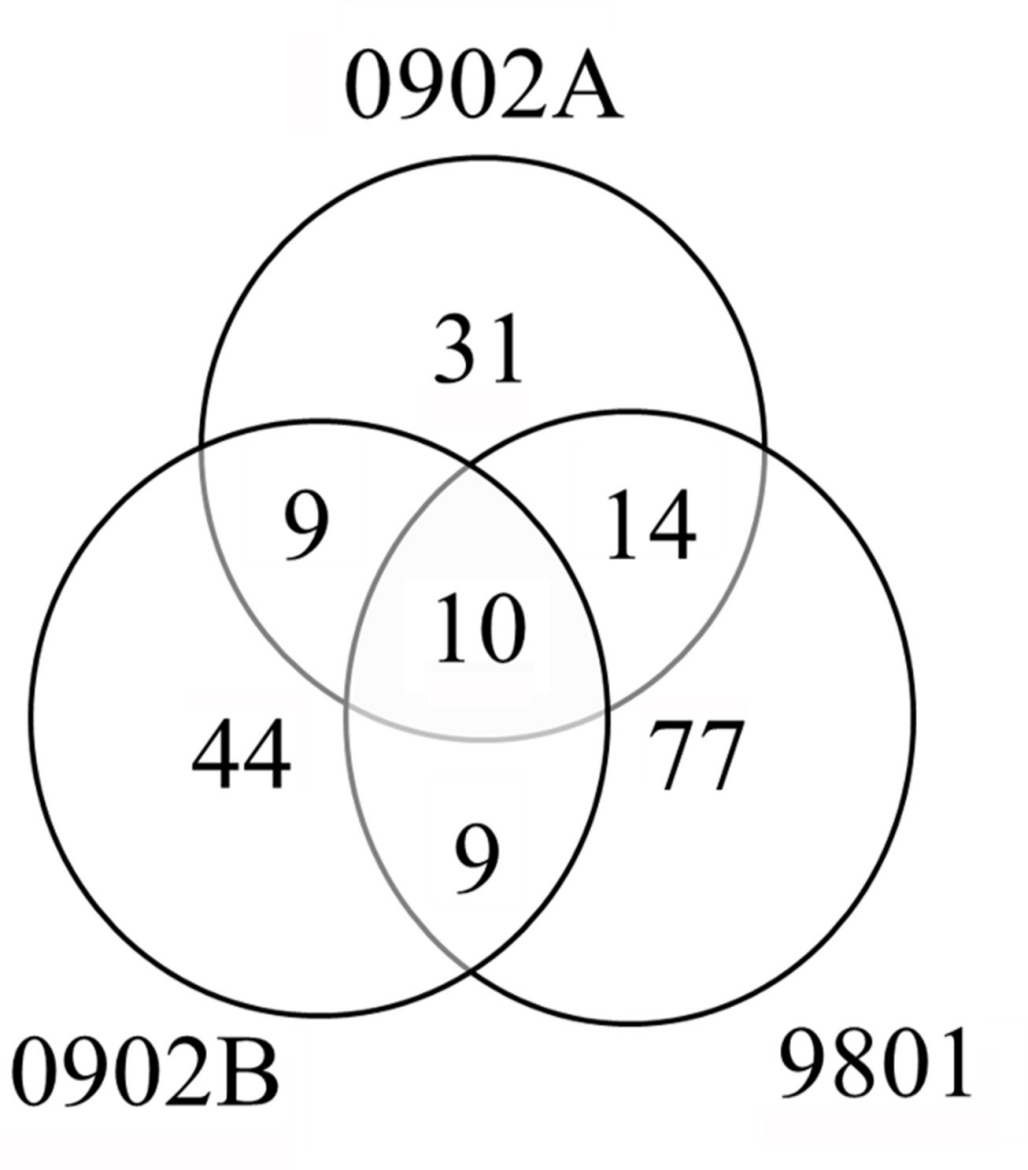
Venn diagram illustrating protein overlaps between PPD 9801, PPD 0902A, and PPD 0902B. The numbers of proteins identified for each PPD are indicated. Although the majority of proteins identified were unique to a given production lot, 10 proteins were present in all three lots analyzed (see S1 Table).

Mass spectrometry analysis was performed on three PPDs, including the reference (9801) and alternative lots (0902A and 0902B). The total number of proteins identified among all three PPDs was 194, 78% of which were unique to an individual PPD (Fig 3). All 194 proteins, including peptide matches and molecular weights are listed in S1 Table. Ten proteins were detected in all three PPD suspensions (Fig 4). These 10 proteins may represent the best candidates for development of a consistent recombinant PPD and are listed at the top of S1 Table. MAP_3840 had the greatest number of peptide matches among all the PPDs, and was also one of the most common proteins observed among PPDs analyzed in the Wynne et al., 2012 and Santema et al., 2009 studies. Furthermore, it was identified as a cell surface protein using a trypsin shaving technique [21]. These results suggest that MAP_3840, which encodes a 70-kDa heat shock protein, is present in high abundance in MAP. Also present among all PPDs were MAP_1588c and MAP_1589c, which are tandemly located on the K-10 genome [22] and have been examined as potential diagnostic antigens long ago [23]. Furthermore, both have been reported as a stress protein [24] and MAP_1589c was also found to be surface located [21].

### Potency Testing of recombinant proteins in guinea pigs

Forty-seven of the 194 proteins identified by mass spectrometry were cloned, expressed and purified in *E*. *coli*. Five of these purified proteins are among the 10 PPD proteins identified from all three preparations. All 47 recombinant proteins are highlighted in red in S1 Table and were used for potency testing to determine if they substantially contribute to the skin test response observed with PPDs. Seven proteins showed reproducible skin test responses that were stronger than or equivalent to the reference lot 9801 (Table 3, highlighted in bold). The strongest skin test response among all recombinant proteins was elicited by MAP_1997, which is annotated as an acyl-carrier protein (Table 3). This protein has not been previously described as an antigen in the literature, although it was listed among surface exposed proteins in MAP [21]. Known antigens that are among these seven proteins include the major membrane protein [25, 26] and the LrpG protein [13, 27]. Seventeen additional proteins produced a measureable skin test response in one of two replicate intradermal inoculations. The MBP/Lac-Z control protein showed no visible reactivity at any inoculation site. Two of the 10 PPD proteins present in all three preparations generated positive skin test responses with MAP_4143 showing the second strongest response.

**Table 3:** Positive skin test responses in a guinea pig potency test of 24 MAP recombinant proteins from 47 total proteins tested.

Protein Description	Locus Tag	Area Measurement (mm^2^)	Standard Deviation^a^
**Acyl carrier protein**	**MAP_1997**	**231.52**	**87.70**
**Elongation factor Tu**	**MAP_4143**	**209.08**	**124.13**
**Electron transfer flavoprotein (Beta-subunit) FixA**	**MAP_3061c**	**192.36**	**87.68**
**Putative uncharacterized protein**	**MAP_2694**	**171.72**	**56.83**
**Putative acyl-CoA dehydrogenase**	**MAP_3651c**	**166.53**	**77.51**
**LprG protein**	**MAP_1138c**	**161.79**	**50.09**
**Major membrane protein 1**	**MAP_2121c**	**128.90**	**11.11**
Peroxisomal multifunctional enzyme type 2	MAP_3567	242.51	N/A
Chaperone protein DnaK	MAP_3840	221.81	N/A
Putative uncharacterized protein	MAP_3692c	148.58	N/A
Lipoprotein LprC	MAP_2497c	141.95	N/A
Superoxide dismutase (Fragment)	MAP_0187c	131.90	N/A
Fructose-bisphosphate aldolase class-I	MAP_4308c	122.33	N/A
LppZ protein	MAP_3041	104.28	N/A
DNA-binding protein HU	MAP_3024c	68.78	N/A
Peptidyl-prolyl cis-trans isomerase	MAP_1693c	48.52	N/A
Fatty acid desaturase	MAP_2698c	44.25	N/A
Antigen 85-B	MAP_1609c	41.96	N/A
ATP synthase subunit alpha	MAP_2453c	41.49	N/A
Serine/threonine protein kinase	MAP_0016c	38.83	N/A
Wag31 protein	MAP_1889c	36.72	N/A
Putative uncharacterized protein	MAP_2020	29.59	N/A
Peroxiredoxin	MAP_1589c	26.12	N/A
6-phosphogluconolactonase	MAP_1174c	16.32	N/A
MBP/Lac-Z	----	0.00	N/A
PPD 9801	----	149.14	10.82
PPD 0902	----	241.84	37.60

^a^ For the proteins which resulted in skin test responses for both inoculation sites the values represent the average response (mm^2^) and the standard deviation from two guinea pigs.

For the 17 proteins, which exhibited only one positive skin test reaction the value represents the response from the single reactive inoculation and the standard deviation is not available (N/A).

Twelve recombinant proteins that showed the largest skin test responses listed in Table 3 were evaluated further in a second guinea pig potency test. Even the four best proteins were still not considered significantly different from the reference 9801 PPD based upon results from the ranking by difference calculations (Table 4). Those recombinant proteins were MAP_1997, MAP_4143, MAP_3567, and MAP_1138c. The only recombinant protein that elicited responses greater than the reference PPD was MAP_1997, but this difference was not significant.

**Table 4:** Guinea pig potency testing of MAP recombinant proteins ranked by subtracted differences.

MAP Protein (Locus tag)	Rec Protein Mean	MAP PPD 9801 Mean	Difference^a^	CI Upper Range^b^	CI Lower Range^b^	Guinea Pig ID’s
MAP_1997	328.93	248.89	80.04	212.00	-51.93	306, 312, 318, 324, 530
MAP_4143	199.68	277.11	-77.43	0.83	-155.69	304, 310, 316, 322, 528
MAP_3567	192.05	272.39	-80.33	88.62	-249.28	305, 311, 317, 323, 529
MAP_1138c	173.70	264.41	-90.72	24.25	-205.68	301, 307, 313, 319, 325
MAP_3061c	106.80	277.11	-170.32	-73.52	-267.12	304, 310, 316, 322, 528
MAP_3651c	72.57	256.51	-183.94	-132.90	-234.99	302, 308, 314, 320, 526
MAP_2121c	74.40	272.39	-197.98	-63.20	-332.77	305, 311, 317, 323, 529
MAP_2497c	50.25	248.89	-198.65	-176.95	-220.34	306, 312, 318, 324, 530
MAP_3692c	34.73	243.82	-209.08	-141.21	-276.95	303, 309, 315, 321, 527
MAP_2694	42.20	256.51	-214.31	-133.99	-294.64	302, 308, 314, 320, 526
MAP_3840	47.95	264.41	-216.46	-92.78	-340.14	301, 307, 313, 319, 325
MAP_0187c	15.31	243.82	-228.51	-184.33	-272.68	303, 309, 315, 321, 527

^a^Differences were calculated by subtracting the MAP protein skin test response from the PPD skin test response for each animal and then averaging the calculated differences for all animals in each treatment group.

^b^Upper and lower range values represent 95% confidence interval (CI) calculations.

### Gamma-interferon Testing using MAP recombinant proteins

**Fig 5:**
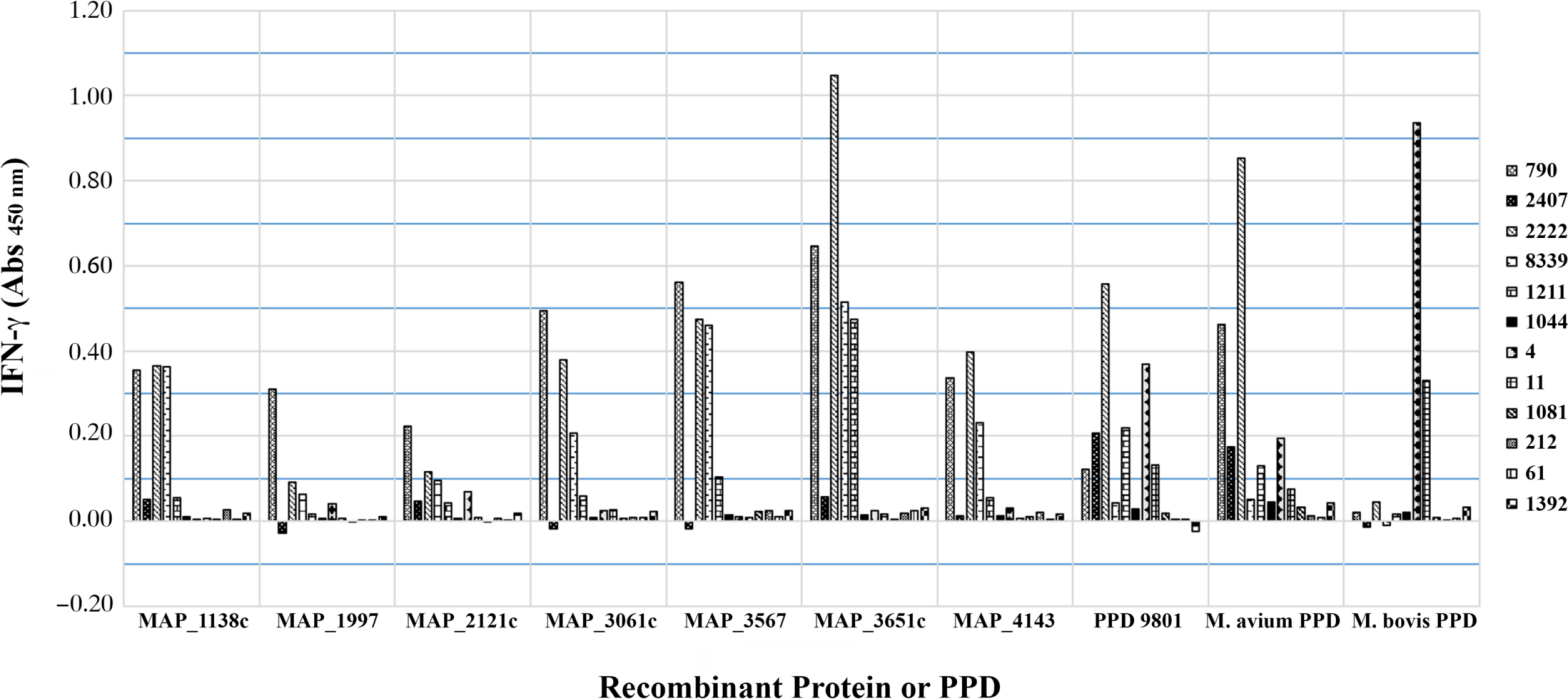
Secretion of IFN-γ using whole blood from Johne’s positive animals, *M*. *bovis* sensitized animals, and negative control animals. Whole blood was incubated with selected recombinant proteins and mycobacterial PPDs for 20–22 hrs as indicated in the materials and methods. Absorbance was measured at 450 nm.

Seven recombinant proteins having the best performance in the guinea pig potency test were further evaluated as mitogens in an IFN-γ stimulation assay. The proteins were evaluated against Johne’s positive animals (#790, #2407, #2222, #8339, #1211, and #1044) based upon results of serological and fecal shedding, *M*. *bovis* sensitized animals (#4 and #11), and negative control animals (#1081, #212, #61, and #1392). MAP_3651c and MAP_3567 demonstrated the greatest IFN-γ stimulation within the Johne’s positive animals with responses in some animals being greater than the stimulation values from MAP PPD 9801 (Fig 5). MAP_1997, which resulted in the greatest skin test response in the guinea pig potency test, showed the least IFN-γ stimulation. Each MAP recombinant protein resulted in negative stimulation values against samples from the *M*. *bovis* sensitized animals (Fig 5). All negative control samples remained negative for IFN-γ stimulation using MAP recombinant proteins or PPD suspensions, indicating good specificity.

In summary, recombinant proteins can be equivalent or a more potent immunological reagent in skin testing than PPD based on guinea pig potency test results. In addition, these recombinant proteins may serve as mitogens in an IFN-γ assay resulting in cytokine stimulation comparable to or greater than that from MAP PPD.

## Discussion

This study had two objectives with the overall goal of analysing a little studied but very important antigen used in Johne’s disease diagnosis and research. One objective was to compare the effect of autoclaving on MAP PPD preparations and measure this affect by SDS-PAGE, immunoblot analysis and guinea pig potency testing. The data collectively suggest that removal of the autoclaving step may enhance antigenicity and prevent protein degradation, but does not negatively affect the potency. A second objective was to define the components present in each preparation through a proteomic analysis and determine proteins commonly present in the different production lots. A subset of the identified PPD proteins was then selected for evaluation in a guinea pig potency test to determine skin test responsiveness in sensitized animals. In addition these proteins were further evaluated to determine their use as antigens in IFN-γ testing. These objectives worked toward the long-term goal of obtaining a more consistent and equally potent PPD that may be composed entirely of recombinant proteins. This reagent could then circumvent the problems inherent in obtaining potent PPDs. Finally, a “recombinant PPD” may avoid cross reactivity issues in cattle vaccinated against *M*. *bovis* or MAP.

The use of high pressure heat inherent in the autoclaving process results in lysis and the appearance of cytosolic proteins that may not be present when autoclaving is eliminated. The presence of the 70-kDa heat shock protein (MAP_3840) in both traditional and alternate PPDs argues against this idea since it is considered a cytosolic protein. However, because this protein is present in high abundance in MAP, it appears at some level in all cell fractions including membrane and secreted fractions [21, 28]. Since secreted or surface proteins should be the first antigenic components encountered in an infected animal, this may allow for more specific responses as well as earlier detection. Multiple studies have shown that many strong antigens are also secreted proteins in MAP [29, 30]. The interest in secreted antigens has primarily involved their significance for use in enzyme-linked immunosorbent assays [28, 31]. In addition, the 10 proteins identified by mass spectrometry and common to all three NVSL PPD preparations were also identified as part of the culture filtrate protein composition characterized by Facciuolo et al [28]. In total there were 58 proteins in the NVSL PPD preparations identified by mass spectrometry that were in common with the Facciuolo et al. study. Furthermore, of the seven recombinant proteins resulting in the greatest skin erythema in the guinea pig potency test, four of those proteins were also identified in the culture filtrate found by Facciuolo et al. [28]. Three of the four proteins, which include MAP_1997, MAP_4143 and MAP_3061c, were also the most reactive in the guinea pig potency test, with MAP_4143 being one of the proteins present in all three PPDs. Therefore, any work toward development of a consistent, recombinant PPD should start with inclusion of MAP_4143 among other proteins.

Potency testing of four MAP PPD lots (9801, 0802, 0803A and 0902) was conducted to ensure that PPDs produced by both methods retained acceptable potency in sensitized guinea pigs. Replacing the autoclaving step with a sterile filtration process would avoid protein degradation, but it was uncertain how this change might affect the potency. Results from potency testing suggest there was no significant effect on potency regardless of whether the culture material was subjected to autoclaving or sterile filtration. This is the first study to examine effect of heating on PPD performance in guinea pig potency testing. However, our results suggest that age of the PPDs might have an effect on potency because the skin test responses where much stronger in the recently made PPDs (0803A and 0902) compared to the reference lot made in 1998. Thus either potency of the new PPDs is significantly better or the age of the reference PPD resulted in decreased potency over time.

Mass spectrometry analysis of PPD preps has revealed several protein identities in these complex protein mixtures. In a separate study, proteomic analysis of *M*. *bovis*, *M*. *avium* subspecies *avium*, and MAP derived PPD suspensions identified 156 proteins among all PPDs [13]. Three of these proteins (MAP_1718c, MAP_3515c, and MAP_1138c) were further analyzed in a serum antibody ELISA with MAP1138c (LprG) emerging as the strongest antigen in high fecal shedding cattle. MAP LprG was also previously identified from a MAP gene fusion library screening study and found to be antigenic in bovine paratuberculosis serum samples by Western blot analysis [32]. Our study also identified LprG and it was among the strongest stimulators in guinea pigs (Table 3). However, LprG showed no significant reactivity in a lymphocyte proliferative assay [13] decreasing its consideration for use in cell-mediated immune response based assays. LprG elicited IFN-γ responses in eight of nine sheep that had been vaccinated with an attenuated MAP strain in the study by Dupont et al. [32]. Using LprG as a mitogen in the IFN-γ assay 50% of Johne’s positive cows, and all *M*. *bovis* sensitized and MAP negative animals were identified correctly, whereas in the Santema et al. [13] study there was no significant difference between positive and negative animals. While results varied for use as an antigen for *in-vitro* IFN-γ stimulation, its ability to elicit immunological responses in both humoral and cell-mediated assays may be indicative that MAP LprG is a highly expressed protein during all stages of Johne’s disease and could prove to be a more versatile candidate for diagnostic assay improvements.

A total of 194 proteins were identified among all preparations with 110 of these identified from lot 9801. This greatly adds to a previous study which identified only six proteins in this same NVSL lot 9801 [14]. It is interesting to note that reference PPD 9801 had the most proteins identified by LC-MS/MS, yet was migrating as a smear in denaturing protein gels and did not appear antigenic by Western blot analysis or as potent by guinea pig sensitization. This shows that while proteins were modified in the heating process, it did not destroy peptides, which are essential for MS identification. Fewer proteins were identified in the alternate PPD lots, with 67 and 74 proteins being identified in each production lot. Although a number of proteins were identified in two of the three preparations analysed, only ten proteins were detected in all three preparations. Combining the results from the Wynne et al. [14] and Santema et al. [13] studies shows the number of proteins consistently appearing in PPD preparations among all three studies is only three. They include MAP_4143, MAP_1595, and MAP_3840. The total number of MAP proteins identified at least once in any of the three studies was 214, which suggests a starting point for the complete PPD proteome of MAP. Although some variability may be attributed to the methodology used for protein identification, the small overlap of proteins nonetheless further suggests the inconsistencies that can be encountered in PPD production processes and the need to improve such processes to reduce or eliminate such variability.

MAP recombinant proteins used as antigens in an IFN-γ assay showed five proteins as potential candidate antigens. MAP_3651c and MAP_3567 were the strongest antigens with Johne’s positive samples and they correctly identified four of the six animals as positive. MAP_3061c, MAP_1138c, and MAP_4143 were comparable to each other, and also identified three of the same four animals identified by MAP_3651c and MAP_3567. These results were comparable to animals identified as positive using MAP PPD 9801 with differences noted for two animals. Cow #2407 was identified as positive by MAP PPD 9801, but was negative by all individual proteins. In contrast, cow #8339 was negative using MAP 9801, but was positive by five of the seven individual proteins. These results were in contrast to the guinea pig potency test results in which MAP_3651c and MAP_3061c did not stimulate skin test responses comparable to MAP PPD 9801. Also in contrast to the strong skin test response in the guinea pig potency test, MAP_1997 was not a strong mitogen in the IFN-γ stimulation assay. These contrasting results between the two tests demonstrate that neither test is a good predictor of success in the other and may be due to the additional immunological responses ongoing at skin test sites that involve more than IFN-γ alone.

There are a number of false positive tuberculin skin test results, particularly in developing countries, due to BCG vaccination and non-tuberculous mycobacteria [33]. The issue of false positive skin tests has been a long-term concern due to the conserved antigenic characteristics of mycobacteria. This situation is similar in the veterinary field with caudal fold tests using *M*. *bovis* PPD and skin tests with MAP PPD giving false positive results if animals are exposed to other mycobacteria. By developing a recombinant PPD-type reagent, it may now be feasible to not only avoid false positive reactions but to have a consistent reagent that could be evaluated in the gamma-interferon assay as well.

## Conclusions

Our results suggest that autoclaving during PPD preparations, though performed for decades, is an unnecessary step in PPD production. Over 100 proteins have now been identified from NVSL PPDs that have been used in field studies for decades. Results from this study have also identified a number of proteins that are reactive to skin testing in sensitized guinea pigs and as antigens in IFN-γ assays. These proteins may contribute to improvements for a well-defined, consistent PPD for diagnostic purposes that may not cross react with TB skin testing. Further research is needed to confirm the DTH responses of these proteins and examine specificity characteristics to differentiate MAP immunological responses from *M*. *bovis* infected animals.

## Supporting Information

S1 Table Proteins identified by mass spectrometry from *Mycobacterium avium* subsp. *paratuberculosis* PPD lot numbers 9801, 0902A, and 0902B. (DOCX)

## References

[1] Garry FWS, Ott S, Hansen D. Info Sheet: APHIS Veterinary Services: Who can afford a $200 loss per cow? OR Johne's disease—What do I need to know? 1999. Available: https://www.aphis.usda.gov/aphis/ourfocus/animalhealth/animal-disease-information/cattle-disease-information/sa_johnes/ct_johnes_disease.

[2] Beard PM, Daniels MJ, Henderson D, Pirie A, Rudge K, Buxton D, et al Paratuberculosis infection of nonruminant wildlife in Scotland. J Clin Microbiol. 2001;39(4):1517–1521. 11283080 10.1128/JCM.39.4.1517-1521.2001 PMC87963

[3] Raizman EA, Wells SJ, Jordan PA, DelGiudice GD, Bey RR. *Mycobacterium avium* subsp. *paratuberculosis* from free-ranging deer and rabbits surrounding Minnesota dairy herds. Can J Vet Res. 2005;69(1):32–38. . 15745220 PMC1142167

[4] Robbe-Austerman S, Gardner IA, Thomsen BV, Morrical DG, Martin BM, Palmer MV, et al Sensitivity and specificity of the agar-gel-immunodiffusion test, ELISA and the skin test for detection of paratuberculosis in United States Midwest sheep populations. Vet Res. 2006;37(4):553–564. Epub 2006/04/28. 10.1051/vetres:2006018 v6035 [pii]. . 16641016

[5] Wren G. Testing for Johne's. Bovine Veterinarian. 1998;(Jul-Aug):4–8.

[6] Billman-Jacobe H, Carrigan M, Cockram F, Corner LA, Gill IJ, Hill JF, et al A comparison of the interferon gamma assay with the absorbed ELISA for the diagnosis of Johne's disease in cattle. Australian Vet J. 1992;69:25–28. 1632724 10.1111/j.1751-0813.1992.tb07426.x

[7] Gwozdz JM, Thompson KG, Murray A, Reichel MP, Manktelow BW, West DM. Comparison of three serological tests and an interferon-gamma assay for the diagnosis of paratuberculosis in experimentally infected sheep. Australian Vet J. 2000;78:779–783. 11194726 10.1111/j.1751-0813.2000.tb10452.x

[8] Kalis C, Collins M, Hesselink J, Barkema H. Specificity of two tests for the early diagnosis of bovine paratuberculosis based on cell-mediated immunity: the Johnin skin test and the gamma interferon assay. Vet Microbiol. 2003;97(1–2):73–86. doi: S0378113503002426 [pii]. . 14637040 10.1016/j.vetmic.2003.07.003

[9] Robbe-Austerman S, Stabel J, Morrical D. Skin test and gamma interferon enzyme-linked immunosorbent assay results in sheep exposed to dead *Mycobacterium avium* subspecies *paratuberculosis* organisms. J Vet Diagn Invest. 2007;19(1):88–90. doi: 19/1/88 [pii]. . 17459838 10.1177/104063870701900114

[10] Stabel JR. Production of gamma-interferon by peripheral blood mononuclear cells: an important diagnostic tool for detection of subclinical paratuberculosis. J Vet Diagn Invest. 1996;8(3):345–350. 8844578 10.1177/104063879600800311

[11] Stabel JR, Whitlock RH. An evaluation of a modified interferon-gamma assay for the detection of paratuberculosis in dairy herds. Vet Immunol Immunopath. 2001;79(1–2):69–81. . 11356251 10.1016/s0165-2427(01)00253-7

[12] Coussens PM. *Mycobacterium paratuberculosis* and the bovine immune system. Animal health research reviews / Conference of Research Workers in Animal Diseases. 2001;2(2):141–161. . 11831436

[13] Santema W, Overdijk M, Barends J, Krijgsveld J, Rutten V, Koets A. Searching for proteins of *Mycobacterium avium* subspecies *paratuberculosis* with diagnostic potential by comparative qualitative proteomic analysis of mycobacterial tuberculins. Vet Microbiol. 2009;138(1–2):191–196. 10.1016/j.vetmic.2009.03.021 . 19349126

[14] Wynne JW, Shiell BJ, Colgrave ML, Vaughan JA, Beddome G, Michalski WP. Production and proteomic characterisation of purified protein derivative from *Mycobacterium avium* subsp. *paratuberculosis*. Proteome Sci. 2012;10(1):22 10.1186/1477-5956-10-22 . 22443541 PMC3337294

[15] Steadham EM, Martin BM, Thoen CO. Production of a *Mycobacterium avium* ssp. *paratuberculosis* purified protein derivative (PPD) and evaluation of potency in guinea pigs. Biologicals. 2002;30(2):93–5. . 12127310 10.1006/biol.2002.0321

[16] Haagsma J, Angus RD. Tuberculin production In: Thoen CO, Steele JH, editors. *Mycobacterium bovis* infection in animals and humans. Ames, Iowa: Iowa State University Press; 1995 p. 73–84.

[17] Bannantine J, Paustian M. Identification of diagnostic proteins in *Mycobacterium avium* subspecies *paratuberculosis* by a whole genome analysis approach. Methods Mol Biol. 2006;345:185–196. doi: 1-59745-143-6:185 [pii] 10.1385/1-59745-143-6:185 . 16957356

[18] Lippolis JD, Brunelle BW, Reinhardt TA, Sacco RE, Nonnecke BJ, Dogan B, et al Proteomic analysis reveals protein expression differences in *Escherichia coli* strains associated with persistent versus transient mastitis. J Proteomics. 2014;108:373–381. 10.1016/j.jprot.2014.06.008 . 24954099

[19] Cho YS, Dobos KM, Prenni J, Yang H, Hess A, Rosenkrands I, et al Deciphering the proteome of the in vivo diagnostic reagent "purified protein derivative" from Mycobacterium tuberculosis. Proteomics. 2012;12(7):979–991. 10.1002/pmic.201100544 . 22522804 PMC3756804

[20] Rennie B, Filion LG, Smart N. Antibody response to a sterile filtered PPD tuberculin in *M*. *bovis* infected and *M*. *bovis* sensitized cattle. BMC Vet Res. 2010;6:50 10.1186/1746-6148-6-50 . 21062483 PMC2994848

[21] He Z, De Buck J. Localization of proteins in the cell wall of *Mycobacterium avium* subsp. *paratuberculosis* K10 by proteomic analysis. Proteome Sci. 2010;8:21 10.1186/1477-5956-8-21 . 20377898 PMC2859856

[22] Li L, Bannantine J, Zhang Q, Amonsin A, May B, Alt D, et al The complete genome sequence of *Mycobacterium avium* subspecies *paratuberculosis*. Proc Natl Acad Sci U S A. 2005;102(35):12344–12349. doi: 0505662102 [pii] 10.1073/pnas.0505662102 16116077 PMC1194940

[23] Olsen I, Tryland M, Wiker HG, Reitan LJ. AhpC, AhpD, and a secreted 14-kilodalton antigen from *Mycobacterium avium* subsp. *paratuberculosis* distinguish between paratuberculosis and bovine tuberculosis in an enzyme-linked immunosorbent assay. Clin Diagn Lab Immunol. 2001;8(4):797–801. 10.1128/CDLI.08.4.797-801.2001 11427429 PMC96145

[24] Gumber S, Taylor DL, Marsh IB, Whittington RJ. Growth pattern and partial proteome of *Mycobacterium avium* subsp. *paratuberculosis* during the stress response to hypoxia and nutrient starvation. Vet Microbiol. 2009;133(4):344–357. 10.1016/j.vetmic.2008.07.021 . 18786786

[25] Bannantine J, Radosevich T, Stabel J, Berger S, Griffin J, Paustian M. Production and characterization of monoclonal antibodies against a major membrane protein of *Mycobacterium avium* subsp. *paratuberculosis*. Clin Vaccine Immunol. 2007;14(3):312–317. doi: CVI.00353-06 [pii] 10.1128/CVI.00353-06 17267586 PMC1828852

[26] Li L, Munir S, Bannantine J, Sreevatsan S, Kanjilal S, Kapur V. Rapid expression of *Mycobacterium avium* subsp. *paratuberculosis* recombinant proteins for antigen discovery. Clin Vaccine Immunol. 2007;14(1):102–105. doi: CVI.00138-06 [pii] 10.1128/CVI.00138-06 . 17079432 PMC1797714

[27] Leite FL, Reinhardt TA, Bannantine JP, Stabel JR. Envelope protein complexes of *Mycobacterium avium* subsp. *paratuberculosis* and their antigenicity. Vet Microbiol. 2015;175(2–4):275–285. 10.1016/j.vetmic.2014.11.009 . 25500374

[28] Facciuolo A, Kelton DF, Mutharia LM. Novel secreted antigens of *Mycobacterium paratuberculosis* as serodiagnostic biomarkers for Johne's disease in cattle. Clin Vaccine Immunol. 2013;20(12):1783–91. 10.1128/CVI.00380-13 . 24089453 PMC3889510

[29] Cho D, Collins MT. Comparison of the proteosomes and antigenicities of secreted and cellular proteins produced by *Mycobacterium paratuberculosis*. Clin Vaccine Immunol. 2006;13(10):1155–61. 10.1128/CVI.00058-06 . 17028217 PMC1595327

[30] Willemsen PT, Westerveen J, Dinkla A, Bakker D, van Zijderveld FG, Thole JE. Secreted antigens of *Mycobacterium avium* subspecies *paratuberculosis* as prominent immune targets. Vet Microbiol. 2006;114(3–4):337–44. 10.1016/j.vetmic.2005.12.005 . 16413703

[31] Shin SJ, Cho D, Collins MT. Diagnosis of bovine paratuberculosis by a novel enzyme-linked immunosorbent assay based on early secreted antigens of Mycobacterium avium subsp. paratuberculosis. Clin Vaccine Immunol. 2008;15(8):1277–81. 10.1128/CVI.00105-08 18550730 PMC2519299

[32] Dupont C, Thompson K, Heuer C, Gicquel B, Murray A. Identification and characterization of an immunogenic 22 kDa exported protein of *Mycobacterium avium* subspecies *paratuberculosis*. J Med Microbiol. 2005;54(Pt 11):1083–1092. . 16192441 10.1099/jmm.0.46163-0

[33] Farhat M, Greenaway C, Pai M, Menzies D. False-positive tuberculin skin tests: what is the absolute effect of BCG and non-tuberculous mycobacteria? Int J Tuberc Lung Dis. 2006;10(11):1192–1204. . 17131776

