# Supplementary material for: Composition and Potency Characterization of Mycobacterium avium subsp. paratuberculosis Purified Protein Derivatives
Source: PLoS One. 2016 May 2;11(5):e0154685. doi: 10.1371/journal.pone.0154685 (PMC4852940; doi:10.1371/journal.pone.0154685)
Supplement: S1 Table — (DOCX) [file pone.0154685.s001.docx]

| S1 Table: Proteins identified by mass spectrometry from *Mycobacterium avium* subsp. *paratuberculosis* PPD lot numbers 9801, 0902A, and 0902B. | | | | | | |
| --- | --- | --- | --- | --- | --- | --- |
|  | | | | Number of Peptide Reads | | |
| **Description** | **Accession** | **MW (kDa)^a^** | **Locus Tag** | **0902A** | **9801** | **0902B** |
| **Chaperone protein DnaK^b^** | **Q00488** | **67** | **MAP_3840** | **106** | **16** | **148** |
| Forkhead-associated protein | A0QGN6 | 17 | MAP_1540 | 8 | 18 | 60 |
| **Peroxiredoxin** | **F7PEC2** | **22** | **MAP_1589c** | **29** | **5** | **29** |
| **Bacterioferritin** | **P45430** | **18** | **MAP_1595** | **10** | **18** | **24** |
| **Elongation factor Tu** | **A0QL35** | **44** | **MAP_4143** | **33** | **18** | **4** |
| Alkyl hydroperoxide reductase AhpD | Q73ZL4 | 19 | MAP_1588c | 10 | 4 | 29 |
| Probable cutinase Cut3 | A0QKI4 | 19 | MAP_3428c | 7 | 4 | 18 |
| Thioredoxin domain protein | A0QCZ3 | 31 | MAP_2435c | 2 | 4 | 6 |
| Polyketide synthase | A0Q9C4 | 193 | MAP_0220 | 4 | 4 | 4 |
| **LpqE protein** | **A0QAB1** | **19** | **MAP_0474c** | **2** | **8** | **3** |
| **Acyl carrier protein** | **A0QER4** | **13** | **MAP_1997** | **33** | **17** |  |
| 60 kDa chaperonin 2 | P42384 | 57 | MAP_3936 | 20 | 29 |  |
| **Major membrane protein** | **A0QED3** | **34** | **MAP_2121c** | **11** | **7** |  |
| **10 kDa chaperonin** | **A0QKR3** | **11** | **MAP_4264** | **9** | **8** |  |
| **ATP synthase - alpha** | **A0QCX6** | **60** | **MAP_2453c** | **2** | **13** |  |
| **Isocitrate lyase** | **A0QGD1** | **85** | **MAP_1643** | **6** | **2** |  |
| Phosphoglycerate kinase | A0QHY4 | 42 | MAP_1165 | 10 | 2 |  |
| **Adenosylhomocysteinase** | **Q73UK6** | **54** | **MAP_3362c** | **14** | **2** |  |
| Glyceraldehyde-3-phosphate dehydrogenase | P94915 | 36 | MAP_1164 | 4 | 8 |  |
| **Fatty acid desaturase** | **F7PAY8** | **31** | **MAP_2698c** | **10** | **2** |  |
| Polyribonucleotide nucleotidyltransferase | A0QIW5 | 80 | MAP_2891c | 7 | 2 |  |
| Nucleoside-diphosphate-sugar epimerase | F7PBR8 | 30 | MAP_0494 | 5 | 2 |  |
| Transcription antitermination protein nusG | A0PM06 | 26 | MAP_4111 | 3 | 3 |  |
| **60 kDa chaperonin 1** | **A0QKR2** | **56** | **MAP_4265** | **2** | **4** |  |
| **LprG protein** | **A0QI11** | **26** | **MAP_1138c** | **7** |  | **32** |
| **Peptidyl-prolyl cis-trans isomerase** | **F7PEP6** | **16** | **MAP_1693c** | **7** |  | **32** |
| **Fructose-bisphosphate aldolase class-I** | **A0QN95** | **32** | **MAP_4308c** | **7** |  | **32** |
| SseA | Q73UG6 | 33 | MAP_3402 | 10 |  | 17 |
| Putative uncharacterized protein | A0QGB4 | 23 | MAP_1659 | 3 |  | 17 |
| Cupin domain protein | A0QIE1 | 12 | MAP_2723c | 3 |  | 14 |
| Chaperone protein ClpB | Q73T66 | 93 | MAP_3853 | 3 |  | 6 |
| Malate synthase G | Q73ZQ2 | 80 | MAP_1549c | 5 |  | 2 |
| Putative uncharacterized protein | A0QLN4 | 14 | MAP_4106 |  | 4 | 35 |
| **6-P-gluconolactonase** | **F7PCY3** | **26** | **MAP_1174c** |  | **3** | **15** |
| Putative uncharacterized protein | A0QLW3 | 16 | MAP_3872 |  | 3 | 13 |
| Elongation factor Ts | P61336 | 29 | MAP_2955c |  | 4 | 11 |
| Adenylate kinase | F7P5P8 | 20 | MAP_4199 |  | 2 | 3 |
| Indole-3-glycerol phosphate synthase | A0QHH0 | 28 | MAP_1305 |  | 2 | 3 |
| **Wag31 protein** | **A0QF61** | **27** | **MAP_1889c** | **14** |  |  |
| **Putative acyl-CoA dehydrogenase** | **A0QME1** | **42** | **MAP_3651c** | **14** |  |  |
| **Protein grpE** | **A0QLZ5** | **24** | **MAP_3841** | **13** |  |  |
| FHA domain-containing protein | F7P1T5 | 55 | MAP_0023c | 6 |  |  |
| **Thiol peroxidase** | **A0QGC1** | **17** | **MAP_1653** | **11** |  |  |
| Citrate synthase | F7P431 | 48 | MAP_0829 | 3 |  |  |
| **Peroxisomal multifunctional enzyme type 2** | **A0QMX5** | **30** | **MAP_3567** | **6** |  |  |
| **HIT domain protein** | **A0QAP1** | **15** | **MAP_0593c** | **8** |  |  |
| Aminopeptidase N | F7PCA9 | 95 | MAP_2287 | 4 |  |  |
| **Electron transfer flavoprotein (Beta-subunit) FixA** | **A0PPW0** | **28** | **MAP_3061c** | **4** |  |  |
| **Antigen 85-C** | **A0QN12** | **38** | **MAP_3531c** | **3** |  |  |
| 3-Hydroxyacyl-CoA dehydrogenase | A0QC92 | 26 | MAP_2637c | 4 |  |  |
| Proteasome subunit beta | A0QFB5 | 31 | MAP_1835c | 4 |  |  |
| **Uncharacterized oxidoreductase MAV_3816** | **A0QJ99** | **30** | **MAP_3007** | **5** |  |  |
| DNA gyrase subunit A | A0Q8S1 | 92 | MAP_0006 | 2 |  |  |
| Alanine--tRNA ligase | A0QI75 | 97 | MAP_1077 | 2 |  |  |
| MoxR protein | A0QHU3 | 42 | MAP_1205 | 3 |  |  |
| **Superoxide dismutase (Fragment)** | **B1A036** | **22** | **MAP_0187c** | **3** |  |  |
| NAD-glutamate dehydrogenase | A0QDD7 | 179 | MAP_2294c | 2 |  |  |
| **Putative uncharacterized protein** | **F7P791** | **47** | **MAP_3692c** | **2** |  |  |
| Putative phosphoserine aminotransferase | Q742L2 | 40 | MAP_0823c | 2 |  |  |
| Biotin-[acetyl-CoA-carboxylase] ligase | A0QKF1 | 27 | MAP_3397c | 2 |  |  |
| **Putative acyl-CoA dehydrogenase** | **A0QPV4** | **44** | **MAP_0150c** | **3** |  |  |
| Succinate-semialdehyde dehydrogenase [NADP(+)] | A0QMB9 | 50 | MAP_3673c | 3 |  |  |
| NAD-dependent aldehyde dehydrogenase | F7P8J8 | 53 | MAP_3413 | 3 |  |  |
| Putative uncharacterized protein | Q745E8 | 17 | MAP_0151c | 3 |  |  |
| DHH family protein | A0QIY0 | 33 | MAP_2905c | 2 |  |  |
| Putative uncharacterized protein | A0QK49 | 47 | MAP_3293 | 2 |  |  |
| hypothetical protein MaviaA2_17504 | UPI0001B59C72 | 43 | MAP_3193 |  | 25 |  |
| **Universal stress protein family protein, putative** | **A0QHD3** | **14** | **MAP_1339** |  | **7** |  |
| ATP synthase subunit beta | A0QCX8 | 53 | MAP_2451c |  | 8 |  |
| p40 protein | A0QME0 | 36 | MAP_3652 |  | 7 |  |
| Pyruvate carboxyltransferase | A0QJU0 | 30 | MAP_3194 |  | 7 |  |
| Electron transfer flavoprotein, alpha subunit | A0QJE9 | 31 | MAP_3060c |  | 6 |  |
| D-3-phosphoglycerate dehydrogenase | A0QJC3 | 54 | MAP_3033c |  | 4 |  |
| YceI like family protein | A0QJU5 | 19 | MAP_3199 |  | 4 |  |
| Fatty acid synthase | A0QD96 | 328 | MAP_2332c |  | 3 |  |
| Cysteine synthase | A0QED1 | 34 | MAP_2123 |  | 3 |  |
| **Transcriptional regulator, Crp/Fnr family protein** | **A0Q9Z7** | **25** | **MAP_0398c** |  | **4** |  |
| **DNA-binding protein HU** | **A0QJB5** | **22** | **MAP_3024c** |  | **3** |  |
| Aminoglycoside phosphotransferase | A0QJU3 | 36 | MAP_3197 |  | 4 |  |
| Chaperone protein htpG | A0QEJ0 | 73 | MAP_2069c |  | 2 |  |
| Trypsin | A0QCH2 | 52 | MAP_2555c |  | 2 |  |
| Alpha oxoglutarate ferredoxin oxidoreductase, beta subunit | A0QDF7 | 39 | MAP_2276c |  | 2 |  |
| Phosphorylase | A0QCZ5 | 96 | MAP_2432c |  | 2 |  |
| Alpha-amylase 3 | A0QGJ0 | 50 | MAP_1587c |  | 2 |  |
| **Antigen 85-B** | **Q06947** | **35** | **MAP_1609c** |  | **3** |  |
| Mycocerosic acid synthase | A0QCC7 | 221 | MAP_2603c |  | 2 |  |
| Putative O-methyltransferase MAV_1364 | A0QCH0 | 23 | MAP_2558 |  | 2 |  |
| Putative uncharacterized protein | A0QFK9 | 37 | MAP_1743c |  | 2 |  |
| NADH-quinone oxidoreductase, chain g | A0QJV3 | 84 | MAP_3207 |  | 2 |  |
| **Virulence factor mvin family protein** | **A0QNC0** | **126** | **MAP_4336** |  | **2** |  |
| Fumarase | F7PAZ3 | 50 | MAP_2693 |  | 2 |  |
| **Putative uncharacterized protein** | **Q73XM1** | **16** | **MAP_2288c** |  | **3** |  |
| Catalase-peroxidase | A0QGA4 | 82 | MAP_1668c |  | 2 |  |
| Putative pterin-4-alpha-carbinolamine dehydratase | P61733 | 10 | MAP_2623 |  |  | 23 |
| Antibiotic biosynthesis monooxygenase domain protein | A0QBF9 | 11 | MAP_0796c |  |  | 19 |
| Putative epimerase, PhzC/PhzF | F7PAJ3 | 24 | MAP_8450 |  |  | 12 |
| **LppZ protein** | **A0QJD0** | **37** | **MAP_3041** |  |  | **15** |
| Cyanate hydratase | A0QL81 | 17 | MAP_4098 |  |  | 12 |
| Antigen 85-C | A0Q9C1 | 31 | MAP_0217 |  |  | 11 |
| Methionine-S-sulfoxide reductase | A0QMY8 | 18 | MAP_3554c |  |  | 10 |
| Deoxyuridine 5'-triphosphate nucleotidohydrolase | A0QIM8 | 16 | MAP_2814c |  |  | 8 |
| Putative uncharacterized protein | A0QAA8 | 28 | MAP_0471 |  |  | 8 |
| Putative uncharacterized protein | Q73VM1 | 15 | MAP_2992c |  |  | 10 |
| 2-amino-4-hydroxy-6-hydroxymethyldihydropteridine pyrophosphokinase | A0QA88 | 19 | MAP_0452 |  |  | 6 |
| **Putative S-adenosyl-L-methionine-dependent methyltransferase MAV_4236** | **A0QKD9** | **32** | **MAP_3385** |  |  | **7** |
| Putative uncharacterized protein | A0Q9Q8 | 14 | MAP_0343 |  |  | 9 |
| **Lipoprotein LprC** | **A0PUQ0** | **20** | **MAP_2497c** |  |  | **10** |
| **Phosphate ABC transporter, phosphate-binding protein PstS** | **A0QBL4** | **37** | **MAP_0872** |  |  | **9** |
| **Cellulose binding domain, putative** | **A0QG62** | **13** | **MAP_1706** |  |  | **8** |
| Peptidyl-prolyl cis-trans isomerase | A0PKC2 | 19 | MAP_0011 |  |  | 2 |
| **Serine/threonine protein kinase** | **A0Q8T1** | **66** | **MAP_0016c** |  |  | **5** |
| Proline-rich 28 kDa antigen | A0Q8W6 | 37 | MAP_0047c |  |  | 6 |
| Acylphosphatase | A0QJ63 | 11 | MAP_2991c |  |  | 5 |
| Ribosome-binding factor A | A0QIY1 | 17 | MAP_2906c |  |  | 4 |
| Putative uncharacterized protein | A0QKI9 | 48 | MAP_3433 |  |  | 5 |
| **N5-carboxyamino-imidazole ribonucleotide mutase** | **A0QKE7** | **18** | **MAP_3393c** |  |  | **3** |
| Nucleoside diphosphate kinase | A0QDG6 | 15 | MAP_2268c |  |  | 4 |
| Putative uncharacterized protein | A0QKY3 | 24 | MAP_4196 |  |  | 2 |
| **Putative uncharacterized protein** | **A0QC28** | **34** | **MAP_2694** |  |  | **4** |
| **Raf kinase inhibitor-like protein, YbhB/YbcL family** | **F7P241** | **18** | **MAP_1885c** |  |  | **2** |
| Putative uncharacterized protein | A1UGX2 | 18 | MAP_1542 |  |  | 2 |
| **Putative uncharacterized protein** | **Q73YD6** | **27** | **MAP_2020** |  |  | **2** |
| Putative uncharacterized protein | A0QBU5 | 60 | MAP_0948 |  |  | 3 |
| Glyoxalase/bleomycin resistance protein/dioxygenase | A0QCP3 | 14 | MAP_2482 |  |  | 3 |
| Glyoxalase family protein | A0Q902 | 16 | MAP_0083c |  |  | 2 |
| **Glyoxalase family protein** | **A0QC51** | **14** | **MAP_2677c** |  |  | **2** |
| Serine esterase, cutinase family protein | A0QKU1 | 29 | MAP_4237c |  |  | 2 |
| Putative uncharacterized protein | A0QLV9 | 21 | MAP_3875c |  |  | 2 |
| **Nudix hydrolase** | **A0QCP9** | **23** | **MAP_2477c** |  |  | **2** |
| Glyoxalase family protein | A0QMB5 | 14 | MAP_3678 |  |  | 2 |
| UPI000203D623 related cluster | UPI000203D623 | 978 | ---- |  |  | 2 |
| CTP synthase | A0PPA8 | 64 | ---- |  | 2 |  |
| FlaB | A0EXH0 | 52 | ---- |  | 13 |  |
| Glyoxalase/bleomycin resistance protein/dioxygenase | A0QCP4 | 15 | ---- |  |  | 3 |
| 30S ribosomal protein S1 | F9QPW7 | 53 | ---- | 2 |  | 3 |
| Uncharacterized protein | F6Q4P8 | 66 | ---- |  | 3 | 3 |
| Putative transcriptional regulator | E8R4J2 | 31 | ---- |  | 3 | 4 |
| Integration host factor MihF | A0PPJ4 | 12 | ---- |  | 15 | 6 |
| Cathelicidin-1 | P22226 | 18 | ---- | 3 |  |  |
| Probable integration host factor | Q0SH36 | 11 | ---- | 7 |  |  |
| Alkyl hydroperoxide reductase C protein AhpC | F5Z3R3 | 22 | ---- | 7 |  |  |
| Alkylhydroperoxide reductase | F9QR33 | 22 | ---- | 8 |  |  |
| GL18924 | B4G7Z2 | 270 | ---- |  | 2 |  |
| Histone H2A | P13912 | 14 | ---- |  | 2 |  |
| Chaperone protein DnaK | A9HEA3 | 67 | ---- |  | 2 |  |
| Glyceraldehyde-3-phosphate dehydrogenase, type I | D5XC94 | 36 | ---- |  | 2 |  |
| Envelope glycoprotein gp160 | E1AD65 | 98 | ---- |  | 2 |  |
| UPI0001CBA8DE related cluster | UPI0001CBA8DE | 40 | ---- |  | 2 |  |
| OSJNBa0053K19.16 protein | Q7XPQ7-R | N/A | ---- |  | 2 |  |
| 2,3-bisphosphoglycerate-independent phosphoglycerate mutase | F7TQM5 | 57 | ---- |  | 2 |  |
| Ketol-acid reductoisomerase | F7TUC4 | 38 | ---- |  | 2 |  |
| Helicase IV | F7TV87-R | N/A | ---- |  | 2 |  |
| Sarcosine oxidase, subunit alpha | F8AFX2 | 51 | ---- |  | 2 |  |
| DNA-directed RNA polymerase | B7DS00 | 135 | ---- |  | 2 |  |
| DNA gyrase subunit A | D5WQZ8 | 90 | ---- |  | 2 |  |
| Ribulose-phosphate 3-epimerase | B3R6Z8 | 25 | ---- |  | 2 |  |
| Lactoferrin | A5HLY3 | 78 | ---- |  | 2 |  |
| UPI00005A5A20 related cluster | UPI00005A5A20-R | N/A | ---- |  | 2 |  |
| ABC multidrug transporter | E0WFL6 | 59 | ---- |  | 2 |  |
| Putative polyketide synthase | B0B505 | 676 | ---- |  | 2 |  |
| Pyridoxal-phosphate-dependent serine hydroxymethyltransferase | A9WI58 | 45 | ---- |  | 2 |  |
| Retrotransposon hot spot (RHS) protein, putative | Q4CLZ8 | 68 | ---- |  | 3 |  |
| UPI0002263A13 related cluster | UPI0002263A13 | N/A | ---- |  | 3 |  |
| ATP synthase F1 sector alpha subunit | F5SH07 | 55 | ---- |  | 3 |  |
| Putative uncharacterized protein | D8TPS7 | 92 | ---- |  | 3 |  |
| Putative uncharacterized protein | D9Y291 | 160 | ---- |  | 3 |  |
| Ubiquitin-conjugating enzyme E2 1 | E5SS96 | 23 | ---- |  | 3 |  |
| Uncharacterized protein | F7DU87 | 27 | ---- |  | 3 |  |
| DNA-binding protein HU | G9QP70 | 10 | ---- |  | 3 |  |
| UPI00016E482A related cluster | UPI00016E482A | N/A | ---- |  | 3 |  |
| ABC transporter ATP-binding protein | F3NCB7 | 34 | ---- |  | 3 |  |
| Putative uncharacterized protein | B6ADN3 | 661 | ---- |  | 3 |  |
| Putative uncharacterized protein | F4CHY3 | N/A | ---- |  | 3 |  |
| Putative multidrug resistance protein | A9FWU2 | 44 | ---- |  | 3 |  |
| Phosphomannomutase | D5UW11 | 48 | ---- |  | 4 |  |
| UPI00021A600D related cluster | UPI00021A600D | 43 | ---- |  | 4 |  |
| 60 kDa chaperonin 2 | D7E1Z7 | 61 | ---- |  | 5 |  |
| 1-pyrroline-5-carboxylate dehydrogenase | C0Z4D9 | 57 | ---- |  | 6 |  |
| Putative cyclohexadienyl dehydratase | C0Z8J3 | 32 | ---- |  | 7 |  |
| hypothetical protein MaviaA2_03487 | UPI0001B59F42 | 122 | ---- |  | 7 |  |
| Elongation factor Tu | A5ZBF7 | 44 | ---- |  | 8 |  |
| Probable oligopeptide ABC transporter substrate binding protein | C0ZAZ6 | 60 | ---- |  | 8 |  |
| Putative uncharacterized protein | E3WQY9 | 40 | ---- |  | 9 |  |
| Elongation factor Tu | D4W4X8 | 43 | ---- |  | 9 |  |
| 50S ribosomal protein L7/L12 | A1T4I8 | 13 | ---- |  | 10 |  |
| S-layer protein | P35825 | 131 | ---- |  | 12 |  |
| ATP synthase subunit beta | C0Z776 | 51 | ---- |  | 17 |  |
| Sensor histidine kinase ChvG | A3S894 | 60 | ---- |  | 22 |  |
| Small acid-soluble spore protein | C0ZH25 | 7 | ---- |  | 63 |  |
| Uncharacterized protein (Fragment) | F7C304 | 49 | ---- |  |  | 2 |
| Alpha amylase catalytic region | D3CVV6 | 91 | ---- |  |  | 2 |
| Chaperone protein DnaK | Q1G1L9 | 67 | ---- |  |  | 2 |
| Uncharacterized protein | F1QN37-R | N/A | ---- |  |  | 3 |
| Putative uncharacterized protein | F8L100 | 351 | ---- |  |  | 6 |

^a^ – MW = molecular weight expressed in kilodaltons. N/A – molecular weight data not available. Proteins are arranged based on presence in the various PPD suspensions.

^b^Proteins in bolded red font were expressed in *E. coli* and tested in this study.
